# Response Monitoring in De Novo Patients with Parkinson's Disease

**DOI:** 10.1371/journal.pone.0004898

**Published:** 2009-03-27

**Authors:** Rita Willemssen, Thomas Müller, Michael Schwarz, Michael Falkenstein, Christian Beste

**Affiliations:** 1 Leibniz Research Centre for Working Environment and Human Factors, WHO Collaborating Research Centre Dortmund, Dortmund, Germany; 2 Department of Neurology, St. Joseph Hospital, Klinik-Weissensee, Berlin, Germany; 3 Department of Neurology, Klinikum Dortmund, Dortmund, Germany; 4 Institute for Cognitive Neuroscience, Biopsychology, Ruhr-University Bochum, Bochum, Germany; University of Groningen, Netherlands

## Abstract

**Background:**

Parkinson's disease (PD) is accompanied by dysfunctions in a variety of cognitive processes. One of these is error processing, which depends upon phasic decreases of medial prefrontal dopaminergic activity. Until now, there is no study evaluating these processes in newly diagnosed, untreated patients with PD (“de novo PD”).

**Methodology/Principal Findings:**

Here we report large changes in performance monitoring processes using event-related potentials (ERPs) in de novo PD-patients. The results suggest that increases in medial frontal dopaminergic activity after an error (Ne) are decreased, relative to age-matched controls. In contrast, neurophysiological processes reflecting general motor response monitoring (Nc) are enhanced in de novo patients.

**Conclusions/Significance:**

It may be hypothesized that the Nc-increase is at costs of dopaminergic activity after an error; on a functional level errors may not always be detected and correct responses sometimes be misinterpreted as errors. This pattern differs from studies examining patients with a longer history of PD and may reflect compensatory processes, frequently occurring in pre-manifest stages of PD. From a clinical point of view the clearly attenuated Ne in the de novo PD patients may prove a useful additional tool for the early diagnosis of basal ganglia dysfunction in PD.

## Introduction

When subjects commit an error in speeded reaction time tasks, a large phasic negative wave with fronto-central midline maximum, called “error negativity” (Ne) [1], or “error related negativity” (ERN) [2], is seen in the electroencephalogram (EEG), which is likely generated in the anterior cingulate cortex (ACC). A recent theory assumes that if an event is worse than expected (i.e. an error), the DA system sends a signal to the anterior cingulate cortex (ACC), which in turn elicits the Ne [3]. DA influx to the prefrontal cortex (PFC) may serve as a gating signal that instructs the network when to maintain a given activity state [4]. Its neuromodulatory effects may strengthen current representations, protecting them against interference from disruption by irrelevant distracting information [4], [5]. In accordance with the dependence of the Ne on the DA-system, the Ne has been shown to be decreased in basal ganglia disorders like Parkinson's (PD), or Huntington's disease (HD) [6]–[8]. Regarding PD, another group [9] found no such reduction in similarly affected PD patients, which has been attributed to possible medication effects. However, it has been shown that medication unlikely affects the modulation of the Ne [10], but the question remains, whether long-term L-dopa medication causes a Ne reduction. Recently, Stemmer et al. [11] found no difference between an early stage PD-group and patients with a long history of medication, also arguing against medication effects on the Ne. Hence the most straightforward approach is to measure the Ne in newly diagnosed patients that are drug-naive, so called “de novo” patients. Analyses in existent studies was restricted to error-related processes, but not on processes related to general response monitoring. Here a component occurring after correct responses (“CRN”) [12] or (“Nc”) [13] is of importance. The Nc has been related to response monitoring [14] or to conflict between the actual response and a response program [15]. Allain et al. [16] have shown that the Nc is reduced in a correct trial preceding an error trial. This supports the monitoring hypothesis and suggests that the Nc is necessary for the maintenance of the proper stimulus-response mapping. Another recent study [13] further suggested that processes reflected by the Nc are generally evident after reactions (reflecting motor response monitoring), and that errors are adding specific processes on these, constituting the Ne [13], [17]. The Nc has occasionally been found to be enhanced in healthy elderly [18], while the Ne has been reported to be reduced in elderly [19]. Similarly, abnormally large Ncs have been observed in patients with PFC-lesions [18] and patients with schizophrenia a disorder known to be associated with PFC dysfunction [20], [21]. According to Coles et al. [22] a damage to prefrontal cortex, or to the pathway from prefrontal cortex to the basal ganglia, is leading to disturbed representations of the correct response and hence to abnormally large Ncs on correct trials. However, the prefrontal cortex has been found to be dysfunctional in PD [23]–[25], which may well affect the Nc, hence leading to abnormally large Ncs in PD.

In the light of a decreased dopaminergic function in older compared to younger as well as in PD-patients compared to healthy controls [26], [27] this may suggest that an error-specific activity (i.e. Ne) protecting a task relevant representation is reduced in its function, while a more general activity, which is evident in correct (Nc) and error trials [13] is enhanced, possibly reflecting a compensatory mechanism. Such a compensatory pattern may be particularly present in newly diagnosed PD patients [28].

In summary the study specifically examines differences in tonic and phasic post-response monitoring processes between de novo PD-patients and healthy controls. Our objective was to test the following hypotheses: first, based on the assumption that the amplitude of the Ne depends on the DA system, we expect that the amplitude of the Ne will be reduced in drug-naive PD patients. Second, the Nc amplitude should be enhanced in de novo PD-patients reflecting the increased overall response monitoring [29] or reflecting the impairment of the correct response representation [22], because prefrontal cortex dysfunctions, frequently observed in PD [23]–[25].

## Materials and Methods

### Subjects

Fourteen newly diagnosed drug-naïve patients with idiopathic PD (7 women) were recruited via the PD out patient unit of the Neurological Clinic, St. Josefs-Hospital, Ruhr-University of Bochum (RUB) and of the Neurological Clinic, Klinikum Dortmund. The mean age of the patients was 59.6 years. Parkinson's disease was diagnosed by means of clinical assessment by the co-authors T.M. and M.S. Subsequently to initial clinical diagnosis all patients were immediately enrolled in the study (between 1 and 3 days after clinical diagnosis). Treatment was postponed until the study protocol (ERP-examination) was completed. To each patient a healthy control subject (N = 14) was matched by age, sex, and educational background. The mean age of the controls was also 59.6 years. None of the control subjects had any history of other neurological or psychiatric disorders, or was taking any drugs affecting the central nervous system. All participants gave signed informed consent after they were informed about the purpose of the study and the protocol was explained to them. The entire study was approved by the ethics committee of the University of Münster. The socio-demographic data of the Ss are given in Tab. 1. All subjects were tested with a battery of standard intelligence and neuropsychological tests in a separate session before the main EEG session. The Multiple Choice Intelligence Test (MWT-B) [30] is a test for crystallized intelligence routinely used in Germany. As a neuropsychological test of executive functioning the Wisconsin Card Sorting Test (WCST) [31] was used. In order to control for depression, the German version of the Beck Depression Inventory (BDI) was carried out [32]. The clinical testing was conducted with the Unified Parkinson's Disease Rating Scale (UPDRS) [33]. The neuropsychological data is given in Table 1, too. In the neuropsychological tests there was no significant difference between the patients and the controls. The overall depression score was relatively low and well below the threshold for depression. However it was higher in the patients (8.3) than in the controls (2.5) (t = 4.3, p<.0001).

**Table 1:** Demographic and clinical characteristics of de novo PD and control subjects.

	de novo PD n = 14		Controls n = 14	
	Mean (SD)	Range	Mean (SD)	Range
Age	58.9 (10.4)	41–75	59 (11.0)	40–74
MWT-B	112 (11.4)	92–130	127 (13.8)	104–130
UPDRS (motor score)	12.5 (5.6)	3–21	N/A	N/A
BDI	8.2 (4.7)	1–17	2.6 (2.4)	0–10
WCST (errors)	34.2 (24.5)	8–84	23.7 (17.3)	8–63
WCST (perseverative errors)	18.2 (19.1)	4–73	11.8 (9.1)	4–33
WCST (categories completed)	4.3 (2.4)	0–6	5.4 (1.5)	1–6

N/A, does not apply. MWT-B, Multiple Choice Intelligence Test; UPDRS, Unified Parkinson's Disease Rating Scale; BDI, Beck Depression Inventory; WCST, Wisconsin Card Sorting Test.

All participants including PD-patients were free of any medication.

### Modified flanker task

The task was originally designed by Kopp et al. [34] and slightly modified for our study. The stimuli consisted of vertical arrays of arrowheads or circles (see Figure S1). The central part of the stimulus was defined as target. When the target was an arrowhead the subjects had to press a button on the side the target pointed to; when the target was a circle, no response had to be given (Nogo trials). Above and below each target a flanker was presented which pointed either to the same side (congruent trials) or to the opposite side (incongruent trials) of the target. Nogo and incongruent trials had a probability of 20% each, congruent trials had a probability of 60%. By making the incongruent stimuli relatively rare we aimed at increasing interference and hence the error rate in the incongruent condition [35]. Right and left pointing flankers were equiprobable. The flankers preceded the targets by 100 ms (Stimulus Onset Asynchrony, SOA = 100 ms) to further strengthen their influence and consequently further increase the error rate in incongruent trials [36]. Flankers and targets were switched off 100 ms after target onset. The next flanker was presented 800 to 1200 ms (interval randomized) after the response of the subjects, or 1900 to 2300 ms after a Nogo target. Altogether 420 stimuli were presented in four blocks of 105 stimuli each, which were interrupted by short breaks. The subjects were asked to react as fast as possible to the arrowhead targets.

A response was given with one of two joystick-like vertical bars. Pressure-sensitive buttons were mounted at the top of the bars and had to be operated with the right and left thumb. Time pressure was administered by an individual deadline and was determined using the error rates in the training session as indicator. A feedback tone (1000 Hz) was presented 500 ms after the response, if the RT was slower than the deadline RT.

### EEG recording and analysis

During task performance the electroencephalogram (EEG) was recorded from 26 electrodes: Fp1, Fpz, Fp2; F7, F3, Fz, F4, F8; FC5, FC3, FCz, FC4, FC6; C3, Cz, C4; P7, P3, Pz, P4, P8; M1, M2; O1, Oz, O2. The vertical EOG was recorded from 4 electrodes above and below both eyes, and the horizontal EOG from 2 electrodes at the outer canthi of the eyes. The amplifier EPA-5 (Sensorium Inc.) was used. The forehead was used as ground. The primary reference was Cz. In addition to EEG and EOG, the response forces of both hands were measured, as outlined above. EEG, EOG and force data were sampled **at** 500 Hz (Acquire, Neuroscan Inc.) and stored continuously on a PC hard-disk together with stimulus and response markers. The data were analyzed off-line using Vision Analyzer (Brain Products, Munich). The EEG was filtered off-line with a filter band-width of 0.5-16 Hz. EEG segments beginning 200 ms before and ending 400 ms after the response were cut out and averaged separately for correct and error responses. The ERP data were re-referenced to average reference to make them independent on any specific reference such as the mastoid. Only the data of the incongruent trials were used for ERP analysis. The Ne in the error trials, and the Nc in the correct trials, were measured as the largest negative peak at FCz within a window of 20 to 120 ms after the response, relative to the baseline.

### Ethics

Parkinson's disease patients were recruited from local clinics, the neurological department of the University of Bochum and the municipal hospital Dortmund. Healthy controls were recruited by newspaper announcements. All participants gave written informed consent. For the Parkinson's disease individuals, a family member was aware of the recruitment for the study and was involved in the consent procedure. The study was approved by the ethics committee of the University of Münster.

### Statistical methods

Data from fourteen de novo PD patients (N = 14) and fourteen healthy controls (N = 14) were analyzed. There were no drop-outs. Reaction times (RTs) and error rates were analyzed as behavioural measures. Neurophysiological processes on correct and erroneous trials were analyzed in a repeated-measures ANOVA using the within-subject factor “correctness” (correct vs. error) and the between-subject factor group (controls, de novo PD). The degrees of freedom were adjusted using the Greenhouse-Geisser-Correction when appropriate. Significances are given one-tailed, due to higher test-power. The mean and standard error of the mean (± SEM) are given. Post-hoc tests were calculated using the Bonferroni-correction. Due to higher test power, one-sided tests were performed. For statistical analysis SPSS 15.0 was used.

## Results

### Behavioral data

Reaction times (RTs) on error and correct trials were subjected to a repeated measures ANOVA with the within-subject factor “correctness” and the between-subject factor “group”. RTs differed between error and correct trials, being significantly longer on correct (512.1±13.4) than on error trials (345.07±12.1) (F(1,26) = 342.78; p<.001). Additionally, there was a main effect “group” (F(1,26) = 6.01; p = .021), showing RTs to be longer in the de novo group (457.9±16.9) than in the control group (399.2±16.9). There was no interaction “correctness×group” (F(1,26) = 0.2; p>.8), indicating that RTs were always longer for correct than for error trials, regardless of group.

For the error rates also a repeated measures ANOVA was calculated using the within-subject factor “trial type” (congruent, incongruent, Nogo) and the between-subject factor “group”. A significant main effect of trial type was obtained (F(2,52) = 50.96; p<.001; η = .66), where it is shown that error rates were lowest on congruent trials (0.52±0.13), followed by Nogo-trials (3.61±0.61) and incongruent trials (13.47±1.75). All trial types differed from each other (p<.001). For the error rates, there was no main effect of group (F(1,26) = 0.02; p>.8) and no interaction “trial type×group” (F(2,52) = 0.16; p>.8). No main effect of slowing was seen (F(1,26) = 1.34; p>.2), which was the case for both groups, as the non-significant interaction reveals (F(1,26) = .09; p>.7).

### Neurophysiological data

Figure S2 shows the response-locked ERPs after correct and incorrect responses for de novo patients and controls at FCz.

A clear Ne is seen for error trials, while the correct trials exhibit a smaller negativity with shorter latency, the Nc. The Ne appears smaller, and the Nc larger in the patients vs. the controls. The difference between Ne and Nc appears very small in the patients. Neurophysiological data were analyzed in a repeated measures ANOVA using the within-subject factor “correctness” and the between-subject factor “group”. The amplitudes of ERPs after error and correct responses differed from each other (F(1,26) = 23.63; p<.001), with the Ne being more negative (−5.79±0.58) than the Nc (−2.17±0.43). While there was no main effect “group” (F(1,26) = .044; p>.5), there was significant interaction “correctness×group” (F(1,26) = 10.89; p = .003). Bonferroni-corrected post-hoc tests revealed that the groups differed on error trials (F(1,26) = 6.28; p = .019), with the Ne being larger in the control group (−7.25±0.82), than in the de novo group (−4.33±0.82). For the Nc the pattern was reversed, as the Nc was larger in the de novo group (−3.16±0.61), compared to controls (−1.19±0.61) (F(1,26) = 5.23; p = .031). For the de novo PDs it is shown that the Ne differed from the Nc (F(1,13) = 5.43; p = .037; η = .29). Yet, in the control group this difference was larger (F(1,13) = 34.92; p<.001; η = .73). Values for the Ne and Nc for each individual patient-control pair are given in Figure S3.

The amplitude of the Ne in the de novo group was unrelated to their RTs in error trials, even though they were prolonged, compared to controls (r<.1; p>.4). For the correct only a trend towards a relation was obtained (r = −.404; p = .066). Correlating the Ne and Nc amplitudes with the BDI score, only revealed significant correlations in the de novo PD group, but not in the controls (Ne: r = −.627; p = .008; Nc: r = −.501; p = .034). The correlation shows that a higher BDI score was related to higher Ne or Nc amplitudes. Yet, in no de novo PD patient the BDI was above the critical cut-off value.

Regarding the latencies, a similar repeated measures ANOVA revealed a main effect “correctness” (F(1,26) = 11.97; p = .002), with the latency of the Ne (74.64±7.62) being longer than the latency of the Nc (43.21±5.09). While the interaction “correctness×group” was significant (F(1,26) = 4.24; p = .049), post-hoc test did not reveal effects (all F's<2.9 p>.1). There was also no main effect “group” (F(1,26) = 1.52; p>.2).

It may be suspected that the Nc waveform is contaminated by residual stimulus-related ERPs. To rule out this possibility, we computed stimulus-locked waveforms on correct trials. As can be seen in Figure S4 amplitudes were not higher in the PD, compared to the control group (F(1,26) = 2.22; p>.15). Hence, even if the Nc is affected by these stimulus-locked ERPs it should have been modulated in the opposite direction, i.e. there should be a reduction of the Nc in de novo-PDs, which was not the case. Thus, the ERP waveforms obtained for the Nc are unlikely to be biased due to differences in stimulus processing.

## Discussion

In the current study we assessed post-response processing functions in recently diagnosed PD-patients, compared to healthy controls. While the Ne was reduced even in the de novo patients, relative to healthy controls the Nc was enhanced in the patients. This pattern cannot be attributed to different performance levels, as the groups did not differ in error rates.

Furthermore, it can be ruled out that the Nc waveform is contaminated by residual stimulus-related ERPs, since the stimulus-locked ERP amplitudes in correct trials were not higher in the PD, compared to the control group. However, the RTs were generally prolonged in the de novo PD-group, which is likely due to the pathogenic mechanisms. In an earlier study [10] the (well-medicated) patients had no prolonged RTs in comparison to matched controls in the same flanker task. This suggests that L-DOPA medication speeds up RT [37]. The prolongation of RTs seems to be unimportant for the modulation of the Ne, as no correlation was found between these parameters. As basic neuropsychological scores did not differ between the groups the results show a clear advantage of neurophysiological measures compared to standard neuropsychological test for detecting early cognitive changes in PD.

The reduction of the Ne in the patient group is in line with the reinforcement-learning hypothesis [3]. In light of this, it is interesting that phasic DA signals in medial frontal areas, as they are reflected in the Ne, are decreased, while neurophysiological processes as they are reflected by the Nc, are enhanced. As hypothesized in the introduction, Nc and Ne may both depend on the activity of the DA-system serving as a gating signal that instructs the network when to maintain a given activity state [4]. This may strengthen current representations, protecting them against interference by irrelevant distracting information [4], [5]. Given this, the results suggest that de novo PD-patients show an increased overall motor response monitoring (Nc) [13] and hence a strengthening of motor response representations. It may be hypothesized that this alteration in medial frontal activity is at costs of error-specific dopaminergic increases: the system controlling motor response monitoring is more demanded in de novo patients, than in controls. If this system is controlled by the DA-system dopaminergic prefrontal neuron assemblies may not be able to alter their firing in order to be capable of the demands that error monitoring processes add on these [13]. In healthy subjects, where dopaminergic neuron assemblies are less strained during motor response monitoring this alteration in firing is possible to a larger extent. Together, these processes may result in a pattern of an increased Nc and a reduced Ne. In our previous studies [8], [10] the Nc was not found to be significantly altered in long-term medicated patients. This pattern of results in de novo PD-patients may be an expression of compensatory processes, which are likely mediated via dopaminergic neurons in PD [38]. However, other studies have not proved the importance of this system [28]. Even though these are predominantly manifest in presymptomatic stages of PD [38] they may persist with reduced efficacy in very early stages of PD (i.e. de novo PD). This pattern of reduced Ne and enhanced Nc resembles what is often seen in healthy elderly vs. young subjects, or in frontal brain patients vs. elderly subjects [18]. Hence a pattern of compensatory enhancement of small DA signals at the cost of strong DA signals after errors appears to exist in normal aging and some CNS diseases. Critical for this interpretation in terms of a general mechanism, may be the finding that the scalp topographies of the Nc and Ne were only similar in the de novo PD group, but not for the controls.

Hence, and more probable the results in the PD-group, may be due to an impairment of the correct response representation that may be due to prefrontal dysfunctions in PD [23]–[25]. Therefore errors are not always detected and correct responses may sometimes be misinterpreted as errors. Such an alternative interpretation has been put forward by Coles et al. [22]. This is also supported by the current data, since the topographies were similar for correct and error trials, but only for the de novo PD group and not for controls. As RTs were prolonged in the de novo group, likely due to a general slowing of motor functions, it may also be hypothesized that this slowing in RTs is an expression of such an increased, overall response monitoring in an early stage of PD. It is possible that the higher BDI score in the de novo patients led to altered response monitoring (Nc) although the Ne amplitude is significantly reduced in the de novo patients, compared to controls.

From a clinical point of view it is highly relevant that the Ne reduction is fairly large in just diagnosed patients which exhibit only subtle signs of manifest PD (UPDRS part III of 12.7). Hence the Ne may have the potential for an additional diagnostic tool in the early diagnosis of PD. Future clinical studies have to show, whether observed modulations of the Ne are specific for various neurodegenerative diseases (e.g. PD, Huntington's disease, supranuclear palsy) as well as other neurological diseases (e.g. multiple sclerosis) and stages within a disease.

Future longitudinal studies may further examine, if the Ne becomes larger and the Nc reduced due to treatment in the course of the disease, suggesting that Ne and Nc may also be useful markers of treatment success.

## Supporting Information

Figure S1 Stimulus arrays of the modified flanker task. Depicted are the stimuli for congruent, incongruent (right hand responses) and for Nogo (no response) condition.
(0.22 MB TIF)

Figure S2 Event-related potentials of the Ne and Nc. The error trials (continuous lines) and correct trials (dashed lines) for de novo PD (dn) (red lines) and controls (green lines) at electrode FCz. R denotes the time of the response. Topographies for error and correct trials separated for the groups are given below.
(0.20 MB TIF)

Figure S3 Scatter plots for the Ne amplitude (upper plot) and Nc amplitude (lower plot) for each individual patient-control pair.
(0.13 MB TIF)

Figure S4 Stimulus-locked ERPs on correct trials for PD patients and controls. Time point “S” denotes the stimulus onset.
(0.87 MB TIF)
