# Supplementary figures and images for: Response Monitoring in De Novo Patients with Parkinson's Disease
Source: PLoS One. 2009 Mar 27;4(3):e4898. doi: 10.1371/journal.pone.0004898 (PMC2657830; doi:10.1371/journal.pone.0004898)

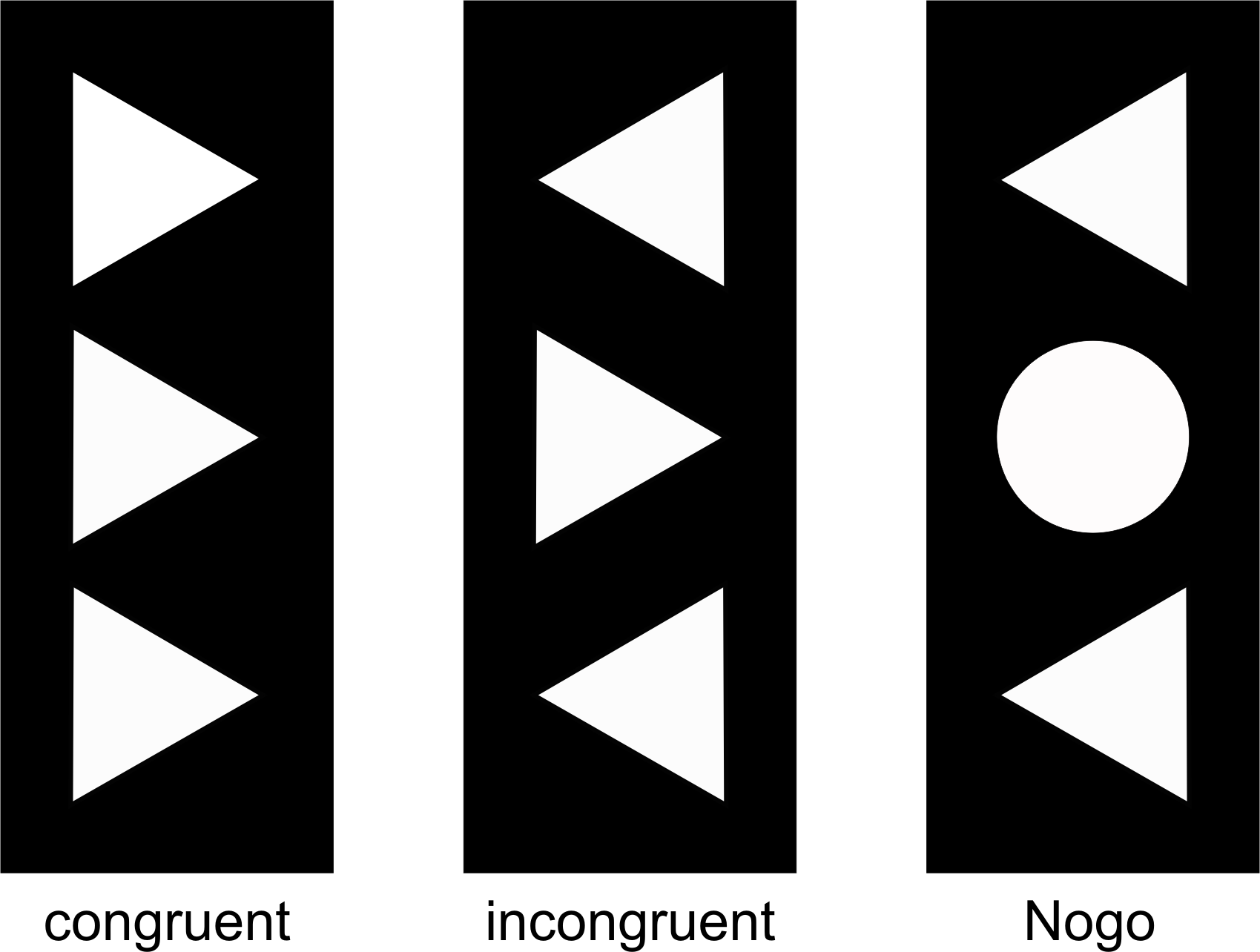

Supplement: Figure S1 — Stimulus arrays of the modified flanker task. Depicted are the stimuli for congruent, incongruent (right hand responses) and for Nogo (no response) condition. (0.22 MB TIF) [file pone.0004898.s001.tif]

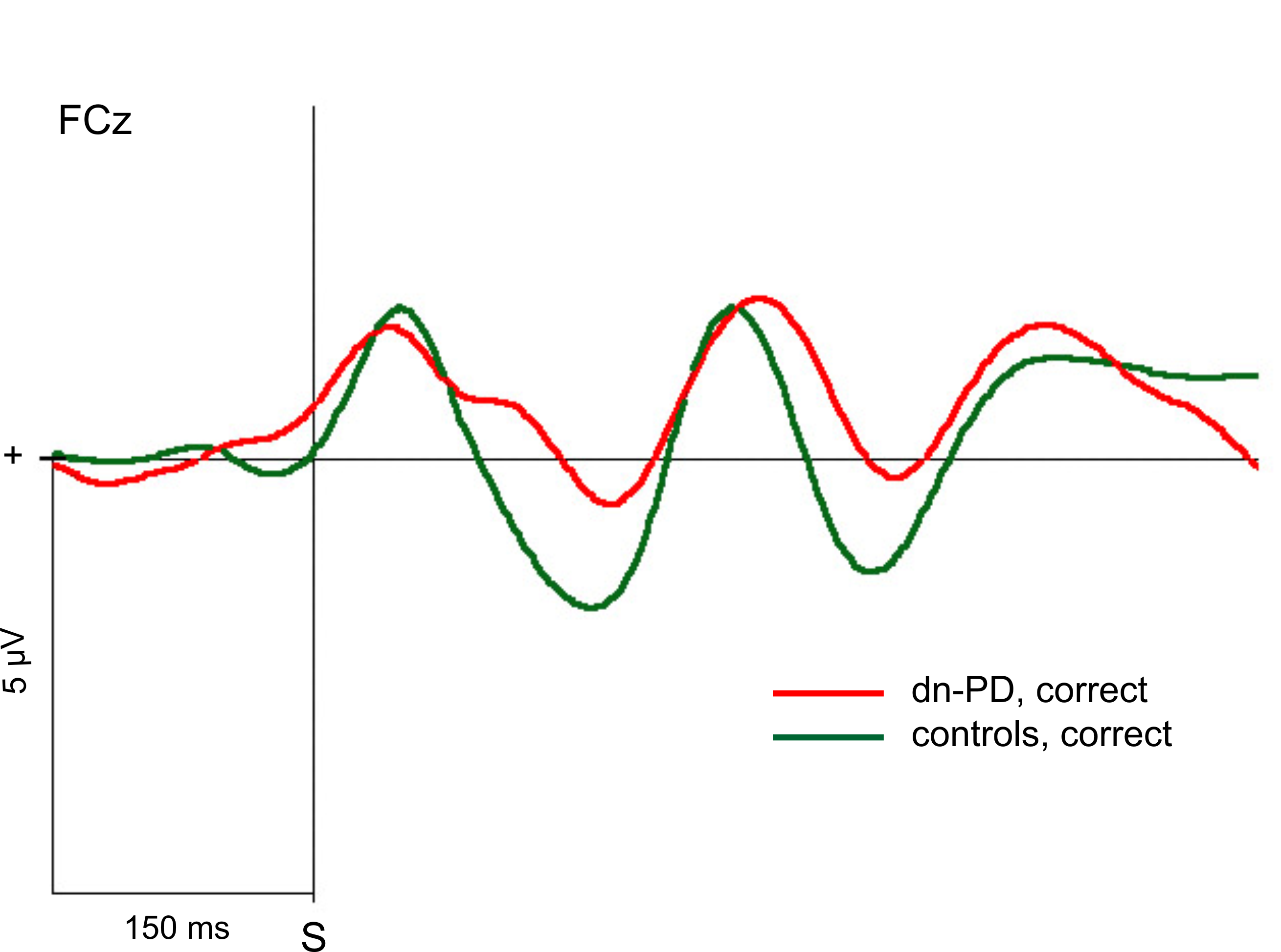

Supplement: Figure S4 — Stimulus-locked ERPs on correct trials for PD patients and controls. Time point “S” denotes the stimulus onset. (0.87 MB TIF) [file pone.0004898.s004.tif]
